# Randomised controlled trial of GM-CSF in critically ill patients with impaired neutrophil phagocytosis

**DOI:** 10.1136/thoraxjnl-2017-211323

**Published:** 2018-07-31

**Authors:** Emma M Pinder, Anthony J Rostron, Thomas P Hellyer, Marie-Helene Ruchaud-Sparagano, Jonathan Scott, James G Macfarlane, Sarah Wiscombe, John D Widdrington, Alistair I Roy, Vanessa C Linnett, Simon V Baudouin, Stephen E Wright, Thomas Chadwick, Tony Fouweather, Jatinder K Juss, Edwin R Chilvers, Susan A Bowett, Jennie Parker, Daniel F McAuley, Andrew Conway Morris, A John Simpson

**Affiliations:** 1 Institute of Cellular Medicine, Newcastle University, Newcastle upon Tyne, UK; 2 Integrated Critical Care Unit, Sunderland Royal Hospital, Sunderland, UK; 3 Intensive Care Unit, Queen Elizabeth Hospital, Gateshead, UK; 4 Intensive Care Unit, Royal Victoria Infirmary, Newcastle upon Tyne, UK; 5 Intensive Care Unit, Freeman Hospital, Newcastle upon Tyne, UK; 6 Institute of Health and Society, Newcastle University, Newcastle upon Tyne, UK; 7 Department of Medicine, University of Cambridge, Cambridge, UK; 8 Newcastle Clinical Trials Unit, Newcastle University, Newcastle upon Tyne, UK; 9 Centre for Experimental Medicine, Queen’s University Belfast, Belfast, UK; 10 Division of Anaesthesia, University of Cambridge, Cambridge, UK

**Keywords:** gm-csf, neutrophil biology, bacterial infection

## Abstract

**Background:**

Critically ill patients with impaired neutrophil phagocytosis have significantly increased risk of nosocomial infection. Granulocyte-macrophage colony-stimulating factor (GM-CSF) improves phagocytosis by neutrophils ex vivo. This study tested the hypothesis that GM-CSF improves neutrophil phagocytosis in critically ill patients in whom phagocytosis is known to be impaired.

**Methods:**

This was a multicentre, phase IIa randomised, placebo-controlled clinical trial. Using a personalised medicine approach, only critically ill patients with impaired neutrophil phagocytosis were included. Patients were randomised 1:1 to subcutaneous GM-CSF (3 μg/kg/day) or placebo, once daily for 4 days. The primary outcome measure was neutrophil phagocytosis 2 days after initiation of GM-CSF. Secondary outcomes included neutrophil phagocytosis over time, neutrophil functions other than phagocytosis, monocyte HLA-DR expression and safety.

**Results:**

Thirty-eight patients were recruited from five intensive care units (17 randomised to GM-CSF). Mean neutrophil phagocytosis at day 2 was 57.2% (SD 13.2%) in the GM-CSF group and 49.8% (13.4%) in the placebo group, p=0.73. The proportion of patients with neutrophil phagocytosis≥50% at day 2, and monocyte HLA-DR, appeared significantly higher in the GM-CSF group. Neutrophil functions other than phagocytosis did not appear significantly different between the groups. The most common adverse event associated with GM-CSF was fever.

**Conclusions:**

GM-CSF did not improve mean neutrophil phagocytosis at day 2, but was safe and appeared to increase the proportion of patients with adequate phagocytosis. The study suggests proof of principle for a pharmacological effect on neutrophil function in a subset of critically ill patients.

**Key messages**

**What is the key question?**

- Acquired impairment of phagocytosis by neutrophils in critically ill patients predicts the development of new infection, and is reversed by granulocyte-macrophage colony-stimulating factor (GM-CSF) ex vivo, but trials have not tested whether GM-CSF restores neutrophil phagocytosis in patients.

**What is the bottom line?**

- GM-CSF did not improve mean neutrophil phagocytosis, but was safe and increased the *proportion* of patients with adequate phagocytosis.

**Why read on?**

- There is a pressing need to develop non-antibiotic-based treatments to reduce infection in critically ill patients, and this ‘personalised medicine’ trial is arguably the first to target patients known to be at highest risk of infection as a consequence of neutrophil dysfunction.

## Introduction

Secondary infections acquired in the intensive care unit (ICU) significantly increase the risk of death in critically ill patients.^1 2^ Over 700 000 hospital-acquired infections are estimated to occur annually in acute care hospitals in the USA,^3^ and infection is more common in ICU than in other hospital areas.

Point prevalence surveys of patients in ICU estimate that approximately 70% are prescribed antibiotics at any time.^4^ High use of antibiotics exerts a selection pressure favouring the emergence of antibiotic-resistant pathogens, which are commonly isolated in ICU-acquired infections (ICUAI).^5^ Furthermore, recent data suggest that overuse of antibiotics in ICUs may be associated with increased mortality.^6 7^ At a time when few effective novel antibiotics are emerging for use in clinical practice,^8^ there is a pressing need to develop non-antibiotic-based strategies for the prevention and treatment of ICUAI.

Neutrophils are the key cellular effectors in clearance of bacterial and fungal infections.^9 10^ Acquired neutrophil dysfunction is common during critical illness. We previously showed that impairment of neutrophil phagocytosis was independently associated with significantly increased risk of ICUAI.^11^ The phagocytic dysfunction is consistently and significantly improved by ex vivo application of granulocyte-macrophage colony-stimulating factor (GM-CSF).^11 12^ GM-CSF is used in clinical practice to stimulate myelopoiesis after administration of chemotherapy, and has been safely administered to critically ill patients.^13–17^

This study therefore sought to test the hypothesis that GM-CSF would improve neutrophil phagocytosis in critically ill patients. Increasing evidence points to the value of identifying treatment-responsive endotypes in clinical trials.^18^ We used a precision medicine approach to target patients with objectively demonstrated impairment of neutrophil phagocytosis, representing those at highest risk of ICUAI.^11^

## Methods

### Patients

Patients were considered eligible if they met all of the following criteria: systemic inflammatory response syndrome on admission to ICU;^19^ recruited within 72 hours of ICU admission; has required support of one or more organ systems (intubation with mechanical ventilation, inotropic support or haemofiltration); expected to survive for at least 48 hours from randomisation (in the opinion of the ICU clinician); and neutrophil phagocytic capacity of <50% (the assay is described below). Exclusion criteria are shown in the online supplemental data section (table S1).

10.1136/thoraxjnl-2017-211323.supp1 Supplementary data

Informed, written consent was obtained from the patient or, where they lacked capacity, from a personal legal representative. When no personal legal representative was available, written consent was obtained from a professional legal representative, under the terms of our ethical approval.

### Randomisation

Patients were randomised 1:1, with stratification by site, using a web-based randomisation service in the Newcastle Clinical Trials Unit. Randomisation was carried out in permuted blocks of variable length.

### Study drug

The study compared recombinant human GM-CSF derived from yeast (Leukine, sargramostim, purchased from Genzyme) reconstituted with sterile water (1 mL sterile water per 250 μg phial of lyophilised GM-CSF), and equivalent volume of placebo (normal saline) according to the patient’s weight. Both appeared as clear, colourless liquids. Research nurses unblinded to allocation administered GM-CSF and placebo by subcutaneous injection. Patients, medical staff, nursing staff providing clinical care and laboratory staff were blinded to allocation. Administration was once daily for 4 days, and injection sites were different every day. The dose of GM-CSF was 3 μg/kg/day.

### Sampling schedule

Whole blood was drawn at baseline (pretreatment), then daily through to day 9 for safety analysis. An extra 25 mL of blood was taken at baseline and on day 2, day 4/5, day 6/7 and day 8/9 for functional assays (neutrophil phagocytosis and other innate immune cellular parameters, as described below).

### Laboratory assays

Blood samples were transported fresh to a single central laboratory (journey time less than 1 hour from each ICU) for analysis by staff proficient in all of the techniques described below. Patients’ neutrophils were isolated from citrated whole blood by dextran sedimentation and discontinuous Percoll gradient.^20^ Neutrophil phagocytosis was quantified as the percentage of neutrophils ingesting ≥2 zymosan particles (derived from the cell wall of the yeast *Saccharomcyes cerevisiae*), which were opsonised using serum from the same patient whose neutrophils were being assessed.^11^ Neutrophils were incubated with serum-opsonised zymosan particles for 30 min and the proportion ingesting two or more particles was determined at light microscopy.

Neutrophil chemotaxis in response to formyl-methionine-leucine-phenylalanine was estimated using the subagarose method,^21^ and superoxide generation using a cytochrome c reduction assay.^22^ Neutrophil apoptosis was measured by flow cytometric analysis of annexin V and propidium iodide in whole blood.^23^ Monocyte HLA-DR expression in whole blood was estimated by flow cytometry using a Quantibrite kit (BD Biosciences, Oxford, UK).^24^ Concentrations of interleukin (IL) 1β, IL-6, IL-8, IL-10, IL-12p70, TNFα and GM-CSF were determined by cytometric bead array (Becton Dickinson). All flow cytometry was carried out using a FACSCanto II flow cytometer (Becton Dickinson).

### Outcome measures

The primary outcome was neutrophil phagocytic capacity 2 days after the first dose of GM-CSF/placebo (measured as the percentage of neutrophils ingesting ≥2 zymosan particles ex vivo).^11^ Secondary outcome measures were serial assessment of neutrophil phagocytosis over 9 days, and quantification of other neutrophil functions such as apoptosis, chemotaxis and superoxide generation. Monocyte HLA-DR expression was also assessed. Clinical and safety outcomes included serial estimation of sequential organ failure assessment (SOFA) Score,^25^ length of stay in ICU, the incidence of ICUAI, all-cause mortality 30 days postrandomisation, and laboratory analyses including full blood count, urea and creatinine, and aminotransferases.

### Safety, serious adverse events (SAEs) and mortality

Safety was monitored by the trial’s data monitoring and ethics committee. Due to the high mortality expected in the critically ill population studied, the trial protocol stipulated that deaths should be included as SAEs only when unexpected and potentially related to study drug.

### Sample size and statistics

Sample size was calculated from previous data analysing the effects of GM-CSF on ex vivo phagocytosis in critically ill patients.^11^ The mean rate of ‘neutrophils ingesting ≥2 zymosan particles' in ICU patients was 39% (SD 13%).^11^ An absolute increase of at least 15% (ie, from 39% to 54% mean phagocytosis) was considered clinically important, on the basis that phagocytosis of ≥50% is associated with less ICUAI, and that phagocytosis rates are rarely less than 30% or greater than 90% using this technique. Therefore, an absolute rise of 15% represents a relative rise of 25% across the range of ‘commonly observed’ phagocytosis rates using our assay. A sample size of 17 in each group gave power of 90% to detect this difference with a significance level of 0.05 using the two-sample t-test. To allow for an attrition rate of approximately 10% we proposed a final sample size of 38 patients (19 per group).

Data were analysed by TC and TF (study statisticians). The study’s statistical analysis plan is found at the end of the online supplemental data section. All authors had access to the primary clinical trial data. We reported descriptive statistics with CIs. The primary end point was analysed initially using a two-sample t-test, and using analysis of covariance in order to allow for the effects of baseline characteristics.

We analysed change from baseline to other secondary time points (day 4/5, day 6/7 and day 8/9) for the primary outcome variable in the same way as the primary end point. Other secondary outcomes were examined using the same methods as for the primary end point.

Fisher’s exact test was used in place of the Χ^2^ test due to the small numbers in the groups. Area under the curve (AUC) was calculated using the linear trapezoidal rule by summing the areas between each time point. In cases where there were missing data with no additional data either before or after the missing time point, we did not calculate the area and treated that patient’s AUC as missing.

Bonferroni’s post hoc correction was used as an informal guide to assess significance or otherwise of the resulting p values.

Primary and secondary outcomes were assessed on an intention-to-treat basis. All analyses were repeated on a per protocol basis for participants who received at least two doses of treatment. SAEs were recorded but not subjected to formal statistical analysis beyond tabulation by group.

Data with missing observations (other than due to mortality) were examined to determine both the extent of, and reason for, such omissions. For the primary outcome measure, there were two missing values. As the missing values are less than 10%, no imputation was carried out.

### Regulatory approvals

The study was registered on publicly accessible databases before the trial commenced (relevant registration numbers are ClinicalTrials.gov NCT01653665, ISRCTN95325384). There was one amendment after the trial commenced but prior to any patient being randomised. This amendment was to undertake a double-blinded rather than a single-blinded trial (reference number 15-09/10/2013).

## Results

**Figure 1:**
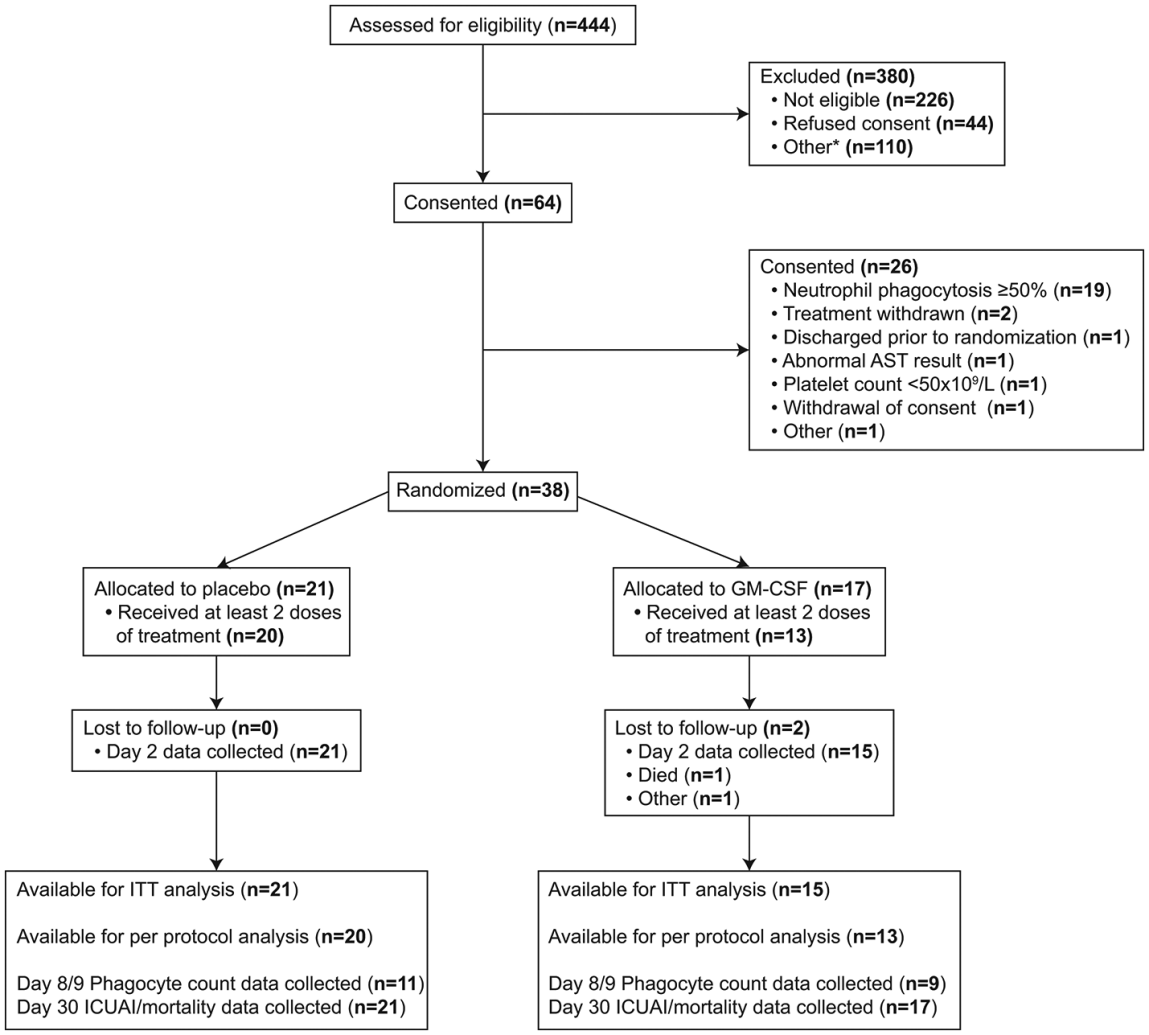
Consolidated Standards of Reporting Trials diagram. AST, aspartate aminotransferase; GM-CSF, granulocyte-macrophage colony-stimulating factor; ICUAI, intensive care unit- acquired infection; ITT, intention-to-treat.

Recruitment ran from November 2013 to February 2015 in five ICUs across four hospitals. The study Consolidated Standards of Reporting Trials diagram is shown in figure 1. All admissions to each ICU were prescreened for eligibility. A total of 444 patients meeting the trial inclusion criteria were screened for eligibility. Of these, 44 patients declined to give consent for participation and 336 were found to be ineligible on the basis of triggering an exclusion criterion or for another reason documented in the online supplemental data section, table S2. Of the 64 eligible patients providing consent, 26 were excluded prior to randomisation, of whom 19 were found to have phagocytic capacity ≥50%. Seventeen patients were randomised to receive GM-CSF and 21 to receive placebo. The imbalance in the allocation ratio was due to the use of block randomisation and stratification.

After an audit of data collection, it became apparent that unblinded nursing staff involved in the administration of the study drug had also undertaken the task of transferring blinded clinical data from source data to the electronic case report form. This had occurred at two of the study sites. Laboratory staff analysing neutrophil outcome measures (including the primary end point) remained blinded at all points and there was no evidence to suggest that this was breached at any stage. On this basis, while the study was intended to be double-blinded, we report it as a single-blinded RCT.

Baseline clinical and demographic features were similar in the two groups (table 1). No patients were neutropenic (neutrophil count of <1×10^9^/L) at baseline. Of the 17 patients randomised to GM-CSF, 4 received less than two doses of study drug (1 died before any administration of study drug and 3 triggered safety criteria for study drug termination (defined in the online supplemental data section, table S3); of these 3, 1 had thrombocytosis before any administration of study drug, 1 developed significant transaminitis after one dose, and 1 developed thrombocytopenia after one dose). Of the 21 patients randomised to placebo, 1 triggered safety criteria for study drug termination (thrombocytopenia after one dose). Therefore, 13 GM-CSF-treated patients received at least two doses of study drug, as compared with 20 in the placebo group.

**Table 1:** Baseline demographic and clinical features

	Placebo	GM-CSF
N	21	17
Median age, years (range)	68 (31–80)	69 (28–89)
Median body mass, kg (range)	82 (45–144)	77 (49–103)
% with a surgical reason for admission to ICU	29	18
Median APACHE II Score (IQR)	21 (18–23)	19.5 (16–27.5)
Sepsis on admission (n)	9	8
Median SOFA Score on admission (IQR)	8 (6–10)	9 (4–11)
Median lowest MAP, mm Hg (IQR)	63 (61–66)	61 (59.5–64.5)

APACHE II, Acute Physiology and Chronic Health Evaluation II; GM-CSF, granulocyte-macrophage colony-stimulating factor; ICU, intensive care unit; SOFA, sequential organ failure assessment; MAP, mean arterial pressure.

There was a small imbalance in neutrophil phagocytosis at baseline, with the mean baseline phagocytosis higher in the group that went on to receive GM-CSF. In the results that follow, the data are presented with adjustment for baseline phagocytosis and site.

**Figure 2:**
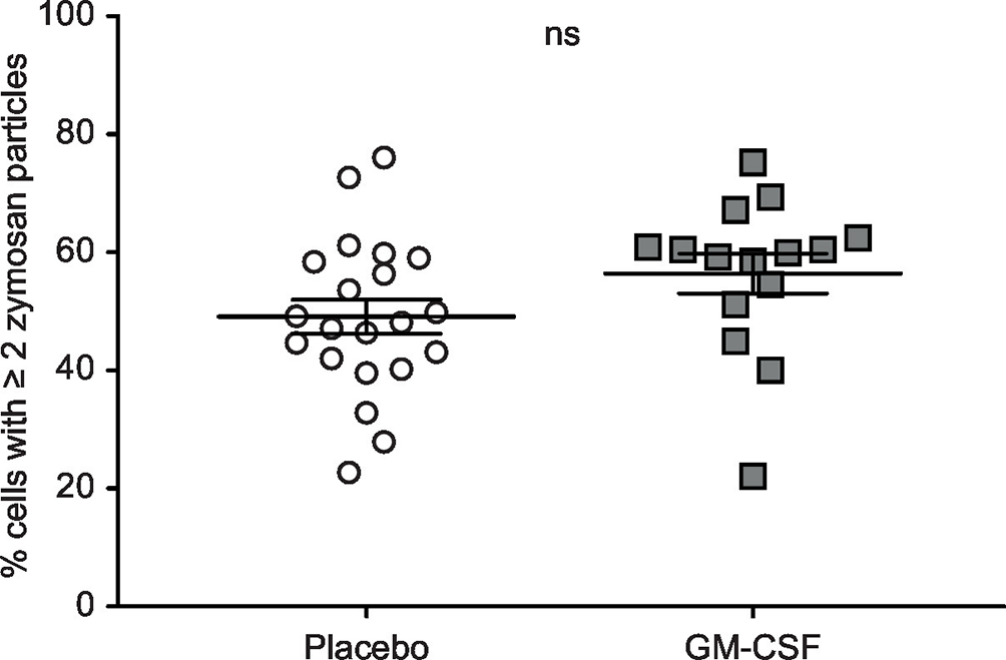
Primary end point: neutrophil phagocytosis at day 2. Neutrophils were isolated from whole blood on day 2 as described in the Methods. Neutrophils were incubated with serum-opsonised zymosan particles for 30 min and the proportion ingesting two or more particles was determined at light microscopy. n=36 (one patient in the GM-CSF group died before day 2, another was withdrawn from the study because of thrombocytosis). Data were analysed on an intention-to-treat basis by two-sample t-test and are shown as individual points. ns, p>0.05. GM-CSF, granulocyte-macrophage colony-stimulating factor.

No significant difference was observed in the primary end point (neutrophil phagocytosis 2 days after the first dose of study drug, p=0.73; figure 2). The proportion of patients with neutrophil phagocytosis ≥50% appeared significantly higher in the GM-CSF group at day 2 and day 6/7, with a similar trend on days 4/5 and 8/9 (table 2).

**Table 2:** Proportion of patients with neutrophil phagocytosis ≥50%

Time point	Proportion of patients with neutrophil phagocytosis ≥50%						P values (Fisher’s exact test for differences)
	Placebo			GM-CSF			
	Number with ≥50%	Number in sample	Per cent ≥50%	Number with ≥50%	Number in sample	Per cent ≥50%	
Day 0	0	21	0%	0	17	0%	No test done
Day 2	9	21	43%	12	15	80%	0.04
Day 4/5	7	16	44%	9	12	75%	0.14
Day 6/7	7	16	44%	10	10	100%	0.004
Day 8/9	7	11	64%	9	9	100%	0.09

GM-CSF, granulocyte-macrophage colony-stimulating factor.

**Figure 3:**
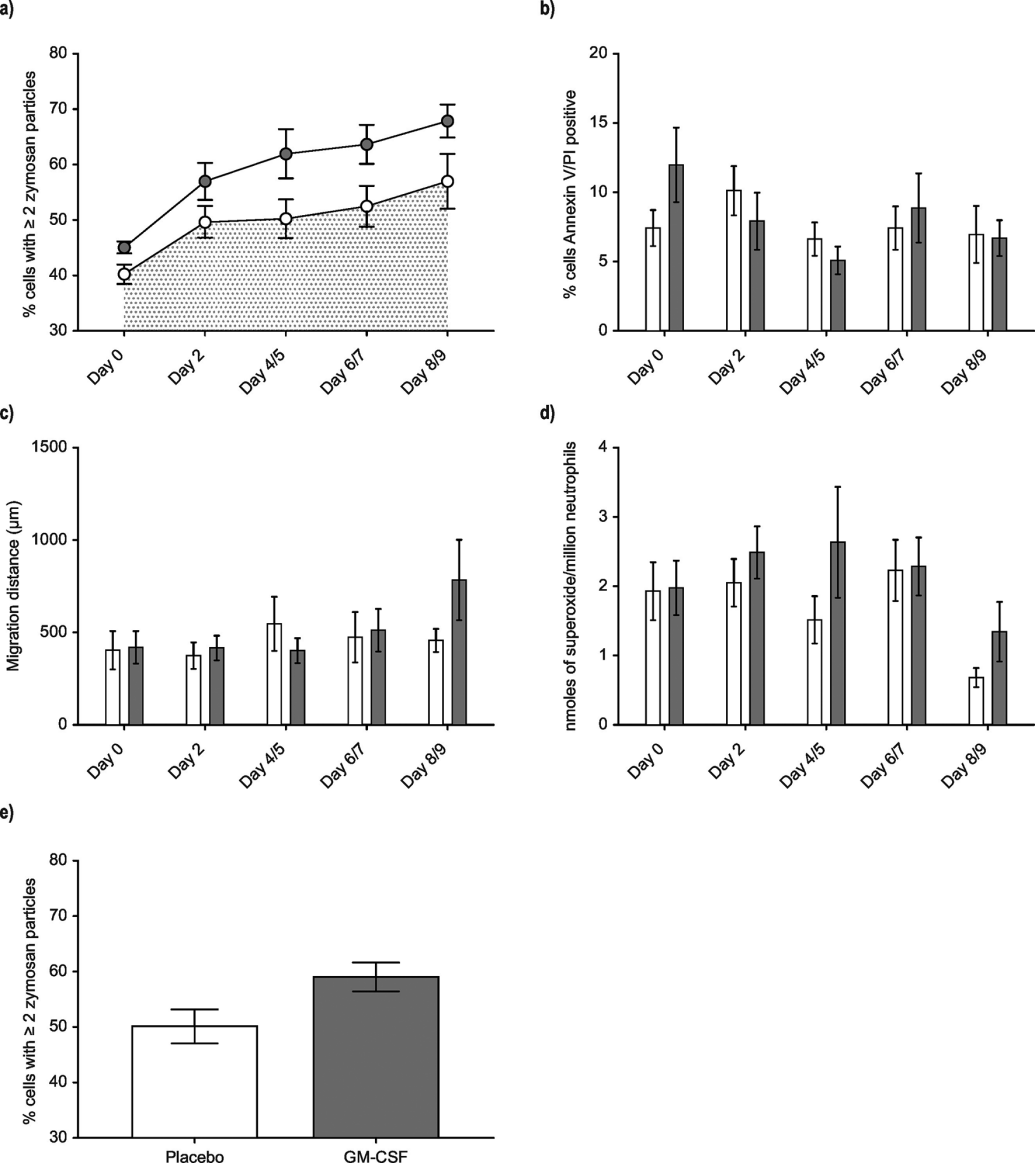
Temporal changes in neutrophil phagocytosis, apoptosis, chemotaxis and superoxide generation. (A) Neutrophils were isolated from whole blood and phagocytosis of serum-opsonised zymosan quantified. Sample sizes (placebo/GM-CSF)—day 0 (21/17); day 2 (21/15); day 4/5 (16/12); day 6/7 (16/10); day 8/9 (11/9). (B) Neutrophil apoptosis was assessed by flow cytometric assessment of annexin V and propidium iodide (PI). Sample sizes (placebo/GM-CSF)—day 0 (17/16); day 2 (17/15); day 4/5 (13/9); day 6/7 (15/10); day 8/9 (8/8). (C) Chemotaxis was assessed using the subagarose method to assess chemotaxis towards formyl-methionine-leucine-phenylalanine. Sample sizes (placebo/GM-CSF)—day 0 (15/12); day 2 (14/12); day 4/5 (9/10); day 6/7 (8/6); day 8/9 (4/3). (D) Superoxide generation was assessed using a cytochrome c reduction assay. Sample sizes (placebo/GM-CSF)—day 0 (18/15); day 2 (17/15); day 4/5 (13/12); day 6/7 (16/10); day 8/9 (11/9). (E) Day 2 phagocytosis was assessed as for (A) above. The data shown are from the per protocol analysis, that is, those patients who received at least two doses of trial drug; n=33 (20 placebo, 13 GM-CSF). All data are shown as mean and SE. In (**A**) open circles=placebo, closed circles=GM-CSF; for (B)–(D), white columns=placebo, dark columns=GM-CSF. For panel (A) statistical comparison of area under the curve for each group used the linear trapezoidal rule by summing the areas between each time point. In cases where there were missing data with no additional data either immediately before or after the missing time point, we did not calculate the area and treated that patient’s area under the curve as missing. For panels (B), (C) and (D) statistical analysis used analysis of covariance (ANCOVA) methods. Bonferroni’s post-hoc correction was used as an informal guide to assess significance or otherwise of the resulting p values. For panel (E) statistical analysis was by unpaired t-test. GM-CSF, granulocyte-macrophage colony-stimulating factor.

The unadjusted AUC for neutrophil phagocytosis at day 9 was greater in the GM-CSF group (figure 3A, which also illustrates the small difference in baseline phagocytosis), but this did not reach statistical significance when adjusted for baseline phagocytosis and site (p=0.14). A 9-day period was used to determine the natural history of neutrophil phagocytosis in the placebo group, and to establish whether any observed effect of GM-CSF was prolonged. No significant differences were apparent in neutrophil apoptosis (figure 3B), neutrophil chemotaxis (figure 3C) or superoxide generation (figure 3D). When the unadjusted primary outcome measure of neutrophil phagocytosis at day two was analysed on a per protocol basis (that is, including only patients receiving at least 2 doses of GM-CSF/placebo), higher neutrophil phagocytosis was found in the GM-CSF group but this did not reach statistical significance (figure 3E) when adjusted for baseline phagocytosis and site (p=0.50).

**Figure 4:**
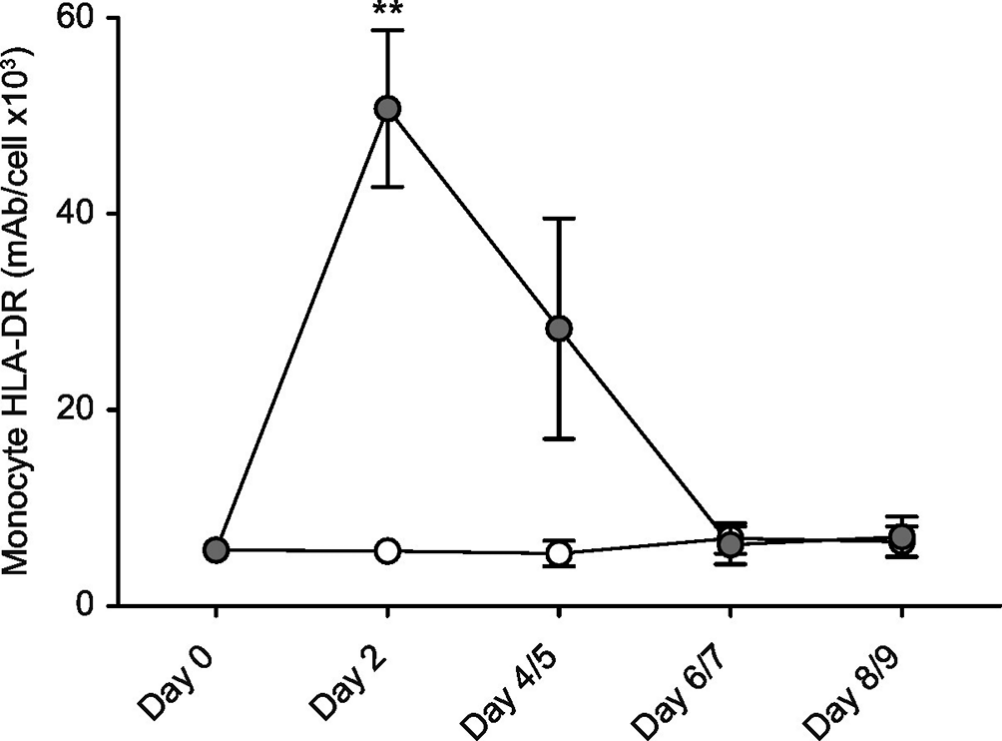
Monocyte HLA-DR. Monocyte HLA-DR was quantified by flow cytometry using a commercial Quantibrite kit. Sample sizes (placebo/GM-CSF)—day 0 (18/16); day 2 (18/13); day 4/5 (14/8); day 6/7 (15/9); day 8/9 (12/8). Open circles=placebo, closed circles=GM-CSF. Data are shown as mean and SE. **, p<0.01. GM-CSF, granulocyte-macrophage colony-stimulating factor; mAb, monoclonal antibodies.

Low monocyte HLA-DR expression has been associated with acquired immunoparesis in critical illness.^26–28^ Monocyte HLA-DR was low at baseline in 26 of the 38 randomised patients. GM-CSF treatment appeared to be associated with a significant rise in monocyte HLA-DR at day 2 (p<0.01, figure 4; the data are shown as individual data points for each time interval in figure S1 in the online supplemental data section).

10.1136/thoraxjnl-2017-211323.supp2 Supplementary data

There were 10 deaths in the 30-day follow-up period of the study (6 in the placebo group, 4 in the GM-CSF group), none of which were considered SAEs. Details relating to deaths are shown in the online supplemental data section (table S4). Three SAEs were described during the trial. One was in the GM-CSF group (intestinal obstruction requiring surgery on day 1, considered unrelated to study drug) and two in the placebo group (one acute respiratory deterioration with pneumomediastinum on day 3; one acute respiratory deterioration with type II respiratory failure on day 4). No suspected unexpected serious adverse reactions (SUSARs) were reported.

Fever was the most common adverse event recorded (four in the GM-CSF group, none in the placebo group). A detailed summary of adverse events is shown in the online supplemental data section (table S5). The SOFA Score was similar in both groups throughout the study period (online supplemental data section (table S6), as were the PaO_2_ to FiO_2_ ratio (online supplemental data section, table S7), full blood count parameters (online supplemental data section, tables S8–10), urea and creatinine (online supplemental data section, table S11 and S12), and transaminases (online supplemental data section, table S13 and S14). Circulating cytokine concentrations are shown in the online data supplement, figure S2.

10.1136/thoraxjnl-2017-211323.supp3 Supplementary data

The trial was not powered to assess differences in infection rates or mortality, and these parameters were analysed on a descriptive basis only. By day 30, 13 ICUAIs developed in six separate patients in the placebo group and 9 ICUAIs in seven separate patients in the GM-CSF group. All-cause 30-day mortality was 6/21 (28.6%) in the placebo group and 4/17 (23.5%) in the GM-CSF group. When considering only patients who received at least two doses of trial drug, all-cause 30-day mortality was 30% in the placebo group (6 of 20 patients) and 7.7% (1 of 13 patients) in the GM-CSF group.

## Discussion

The novelty of this study lies in only including patients whose neutrophil phagocytosis was known to be impaired (representing a group at particularly high risk of ICUAI). This cohort included a broad spectrum of patients with causes for admission representative of ICU case mixes. Other groups studying potential immunomodulatory effects of GM-CSF in critical illness have concentrated largely on patients with sepsis.^13–16^

No statistically significant difference was observed in neutrophil phagocytosis 2 days after the first dose of GM-CSF by intention-to-treat. However, we believe that the apparent significant differences in the proportion of patients with phagocytosis ≥50% suggest an important biological effect of GM-CSF on neutrophil phagocytosis in a subset of patients (table 2). We chose the zymosan assay to assess neutrophil phagocytosis (and a cut-off of 50% for inclusion in the trial) because of our previous experience with the assay which, to the best of our knowledge, is the only neutrophil phagocytosis assay shown to predict for ICUAI independently in critically ill patients.^11^ Reassuringly, using this assay, the frequency of patients consented who had phagocytosis <50% (and therefore went on to randomisation) was similar to that in our previous work, which studied a similar cohort of patients in a different institution. Neutrophil phagocytosis is functionally associated with neutrophil CD88 expression,^11 12^ and CD88 levels (as a surrogate for phagocytosis) have been shown to be predictive for ICUAI.^29^

Our findings of higher neutrophil phagocytosis over time (figure 3A) in the GM-CSF group and of higher phagocytosis at day 2 in patients receiving at least two doses of trial drug (figure 3E) were both confounded by an unexpected difference in baseline neutrophil phagocytosis pretreatment, and were not statistically significantly different. However, we know of no biological reason or data to suggest that the effect of GM-CSF on neutrophils should be influenced by baseline phagocytosis. Indeed, GM-CSF increases phagocytosis in vitro regardless of baseline levels. Furthermore, despite the small difference at baseline, the rate of recovery of neutrophil phagocytosis appears greater over the 4 days of GM-CSF administration, as evidenced by the steepness of the slopes in figure 3A. The consistent direction of effect in the GM-CSF-treated group supports our cautious conclusion that there is a small but true biological response of GM-CSF on neutrophil function in vivo. To our knowledge this is the first demonstration that neutrophil phagocytosis can potentially be improved specifically in those critically ill patients in whom it is impaired, though clearly these findings require to be validated, and the proportion of patients likely to benefit most from GM-CSF requires to be carefully defined.

Enhanced neutrophil phagocytosis and the striking improvement in monocyte HLA-DR (figure 4) suggest combined beneficial immunomodulatory effects for GM-CSF in vivo. The close temporal relationship between GM-CSF administration and HLA-DR expression provides strong evidence, in our view, that GM-CSF was absorbed and biologically active. Similar GM-CSF blood concentrations in both groups (online supplementary figure S2) do not contradict this view, as blood sampling for cytokine estimations was performed almost a day after GM-CSF administration, and blood concentrations of GM-CSF generally return to pretreatment levels within 6–20 hours.^30 31^ HLA-DR is a good marker of immunoparesis,^26^ and GM-CSF has been shown to improve HLA-DR in critical illness.^13 16^ However, effects of GM-CSF on immunomodulatory monocyte *functions* have not been demonstrated in vivo in humans, and were not the focus of this study.

GM-CSF appeared to be safe in the cohort studied. GM-CSF delays neutrophil apoptosis, promotes adhesion to pulmonary endothelium in vitro, and prolongs neutrophil retention in the human lung,^32–34^ leading to the theoretical concern that it may promote acute respiratory distress syndrome in already critically ill patients. However this has been countered by data from other studies.^35 36^ We found no effect of GM-CSF on PaO_2_:FiO_2_ ratios (online supplemental data section, table S7). Similarly we observed no effect of GM-CSF on apoptosis, chemotaxis or extracellular superoxide generation (figure 3). The most common adverse event associated with GM-CSF was fever, though our small sample size may not have allowed the detection of uncommon adverse effects.

We believe the strengths of this study lie in the ‘precision medicine’ approach of including patients at the greatest risk of ICUAI, the focus on a relevant innate immune function (rather than a biomarker), and the objective rigour of a multicentre, randomised, placebo-controlled trial in a relatively understudied population. However, we recognise that the study also has several potential limitations.

First, and most importantly, while we intended this study to be double-blinded, the retrospective discovery of a potential breach in blinding, related to the recording of clinical data, resulted in the study being reported as single-blinded. However we are unaware of any possibility that laboratory staff could have been unblinded in performing and reporting neutrophil assays.

Second, the number of patients recruited was relatively small. Based on our previous studies, we had not anticipated the rate of recovery in mean phagocytosis observed in the placebo group (figure 3A). As a consequence the study may have been underpowered to detect a significant difference in the primary end point.

The small sample size also probably explains the unexpected imbalance in mean neutrophil phagocytosis at baseline. In retrospect it could be argued that the primary end point for the study should have been the proportion of patients with phagocytosis ≥50%, given that phagocytosis at this level appears to confer protection against ICUAI.

A further limitation relates to the number of patients screened relative to the number recruited (figure 1). This reflected the requirement to make patient safety the absolute priority in a setting where an investigational medicinal product is being given to already critically ill patients. Our exclusion criteria were deliberately stringent, and were based on a range of potential adverse events associated with GM-CSF. The strict exclusion criteria and high screen to recruitment ratio clearly lead to questions over how generalisable the data are. However, it is perhaps reassuring that the frequency of neutrophil dysfunction and monocyte HLA-DR suppression were closely comparable to those described in our previous work.^24^ Our safety data, along with those from other studies of GM-CSF in critical illness, suggest that the stringency of exclusion criteria could be relaxed in future studies.

Finally, the assay used to test neutrophil phagocytosis has the limitation that it is labour-intensive and operator-dependent, and its widespread use in hospital laboratories is hard to envisage. Importantly, the assay was chosen specifically because it predicts ICUAI in our hands, while automated assays have not been shown to do so thus far. Interobserver variation in the assay among the experienced operators in our lab was −1.04% (95% CI −2.82% to 0.73%). The test was therefore ideal for our purposes but would be harder to implement in larger studies.

In summary, this study supports a beneficial effect of GM-CSF on monocyte HLA-DR, and suggests a beneficial effect on neutrophil phagocytosis, at least in the *proportion* of patients responding. As low neutrophil phagocytosis and monocyte HLA-DR both predict ICUAI, and given the requirement to develop non-antibiotic-based strategies for prevention of ICUAI, we believe GM-CSF warrants evaluation in further clinical studies, either alone (with or without assessment of higher doses), or in combination with other immune stimulants.
