# Supplementary material for: Randomised controlled trial of GM-CSF in critically ill patients with impaired neutrophil phagocytosis
Source: Thorax. 2018 Jul 31;73(10):918–25. doi: 10.1136/thoraxjnl-2017-211323 (PMC6166597; doi:10.1136/thoraxjnl-2017-211323)
Supplement: Supplementary data [file thoraxjnl-2017-211323supp001.pdf]

SUPPLEMENTAL DATA

Randomised controlled trial of GM-CSF in critically ill patients with impaired neutrophil phagocytosis

EM Pinder, AJ Rostron et al

SUPPLEMENTARY TABLES

Table S1. Exclusion criteria.

- Absence/refusal of informed consent
- Current prescription of a colony-stimulating factor
- History of allergy/adverse reaction to GM-CSF
- Total white cell count >30x10^9/L at the time of screening
- Haemoglobin < 7.5g/dL at the time of screening
- Age < 18 years
- Pregnancy or lactation
- Known in-born errors of neutrophil metabolism
- Known haematological malignancy and/or known to have >10% peripheral blood blast cells
- Known aplastic anaemia or pancytopenia
- Platelet count <50x10^9/L
- Chemotherapy or radiotherapy within the last 24 hours
- Solid organ or bone marrow transplantation; use of maintenance immunosuppressive drugs other than maintenance corticosteroids (allowed up to 10mg prednisolone/day or equivalent)
- Known HIV infection
- Active connective tissue disease (e.g. rheumatoid disease, systemic lupus erythematosus) requiring active pharmacological treatment
- ST-segment elevation myocardial infarction, acute pericarditis (by electrocardiographic criteria) or pulmonary embolism (radiologically confirmed) in the previous week
- Involvement in any study involving an investigational medicinal product in the previous 30 days

Table S2. Reasons for exclusion up to the point of obtaining consent.

**Satisfied exclusion criteria in Supplemental Table S1**	**n**
Absence /refusal of informed consent	44
Current prescription of a colony-stimulating factor	6
History of allergy / adverse reaction to GM-CSF	5
Total white cell count > $30x10^9$/L at the time of screening	20
Haemoglobin < 7.5g/dL at the time of screening	19
Age < 18 years	7
Pregnancy or lactation	9
Known in-born error of neutrophil metabolism	4
Known haematological malignancy and / or known to have > 10% peripheral blood blast cells	14
Known aplastic anaemia or pancytopenia	3
Platelet count < $50x10^9$/L	24
Chemotherapy or radiotherapy within the last 24 hours	10
Solid organ or bone marrow transplantation	22
Use of maintenance immunosuppressive drugs other than maintenance corticosteroids (allowed up to 10mg prednisolone/day or equivalent)	14
Known HIV infection	10
Active connective tissue disease (e.g. rheumatoid disease, systemic lupus erythematosus) requiring active pharmacological treatment	5
ST segment elevation myocardial infarction, acute pericarditis (by ECG criteria) or pulmonary embolism (radiologically confirmed) in previous week	32
Involvement in any study involving an investigational medicinal product in the previous 30 days	13
Any combination of above	9
**Other**	
Due for discharge from critical care on day of screening	45
ICU clinical consultant decided participation not appropriate	13
Specialty clinician not in agreement with patient's participation	35
No laboratory research personnel available	7
Patient previously enrolled	1
Frequent refusal of treatment by patient	1
Other	8
**TOTAL**	**380**

Table S3. Study drug termination criteria.

- maximum treatment period (4 daily doses)
- study drug-related serious adverse reaction
- discharge from a critical care environment
- death
- discontinuation of active medical treatment
- the patient, personal legal representative or professional legal representative requests withdrawal from the study
- decision by the attending clinician that the study drug should be discontinued on safety grounds.

In the following situations consideration will be given to either discontinuing the study drug or reducing the dose by half to minimize the risk of complications

- white blood count > 50,000 cells/mm^3
- platelet count > 500,000 cells/mm^3

Table S4. Deaths.

Group	Time from randomisation to death (days)	Comments/cause of death
Placebo	14	Multi-organ failure
Placebo	4	Refractory septic shock
Placebo	7	Sepsis secondary to small bowel perforation
Placebo	29	Long-term post-operative complications of emergency nephrectomy
Placebo	2	Sepsis, stroke, type II respiratory failure
Placebo	3	Pneumonia and cardiac failure
GM-CSF*	10	Pneumonia, multiple comorbidities
GM-CSF**	1	Large bowel obstruction, multiple comorbidities
GM-CSF	5	Chronic obstructive pulmonary disease, type II respiratory failure, acute kidney injury
GM-CSF***	5	Biliary sepsis

*, only one dose administered because of transaminitis on day 1
**, only one dose administered
***, only one dose administered due to thrombocytopenia.

Table S5. Adverse events.

	Placebo			GM-CSF		
Day	Mild	Moderate	Severe	Mild	Moderate	Severe
0	1. Internal jugular vein thrombus	None	None	None	None	None
1	1. Thrombo-cytopenia	None	None	1. Fever	1. Thrombo-cytopenia.<br><br>2. Raised hepatic transaminases	1. Bowel obstruction with clinical deterioration
2	None	None	None	1. Fever<br><br>2. Thrombo-cytopenia	None	None
3	None	None	1. Pneumo-mediastinum and bilateral consolidation	1. Fever,<br><br>2. Raised platelet count<br><br>3. Raised hepatic trans-aminases	None	None
4	None	None	1. Sudden deterioration with type 2 respiratory failure of unknown cause	1. Fever	None	None
5	None	None	None	None	None	None
6	1. Hypo-glycaemia	1. Increased oxygen require-ments	None	None	None	None
7	None	None	None	1. Internal jugular vein thrombus at central vein catheter insertion site.	None	None
8	None	None	None	None	None	None
9	None	None	None	1. Raised hepatic trans-aminases	None	None

Table S6. SOFA scores for all patients who remained on the ICU.

	Sequential Organ Failure Assessment (SOFA)									
	Day 0	Day 1	Day 2	Day 3	Day 4	Day 5	Day 6	Day 7	Day 8	Day 9
**GM-CSF**										
n	16	16	15	14	14	13	11	10	10	9
median	9	9.5	7	6	8	6	6	7	6	6
range	(3,19)	(2,15)	(2,15)	(0,13)	(0,14)	(0,14)	(0,14)	(0,14)	(0,14)	(0,13)
IQR	(4,10.5)	(5,13)	(2,11)	(4,10)	(2,9)	(3,10)	(2,9)	(3,8)	(2,9)	(3,9)
**Placebo**										
n	21	21	21	19	19	15	15	14	11	14
median	8	7	7	6	5	6	5	4	5	4.5
range	(1,13)	(0,14)	(0,14)	(0,13)	(2,10)	(1,10)	(0,12)	(0,15)	(1,15)	(0,12)
IQR	(6,10)	(6,11)	(5,9)	(4,10)	(3,9)	(3,8)	(2,10)	(1,9)	(2,12)	(1,8)

Table S7. PaO_2:FiO_2 ratios. For all intubated and mechanically ventilated patients the lowest and highest PaO_2:FiO_2 ratios on a given day were recorded. All PaO_2:FiO_2 values are in kPa.

		Placebo		GM-CSF	
Day		Lowest ratio	Highest ratio	Lowest ratio	Highest ratio
0	n	20	20	16	15
	mean (SD)	27.4 (13.2)	38.3 (17.7)	23.0 (11.0)	33.9 (14.9)
	range	(7.6, 54.5)	(11.4, 65.0)	(9.9, 49.5)	(13.2, 59.1)
1	n	20	20	16	16
	mean (SD)	28.5 (13.5)	42.5 (19.4)	21.4 (10.8)	38.3 (14.3)
	range	(7.9, 52.8)	(9.2, 91.3)	(8.3, 52.4)	(18.0, 65.5)
2	n	20	20	14	14
	mean (SD)	27.3 (12.7)	38.8 (15.6)	27.6 (9.7)	37.0 (15.3)
	range	(6.7, 50.0)	(10.6, 61.4)	(10.9, 43.8)	(16.5, 68.5)
3	n	19	19	13	13
	mean (SD)	26.3 (13.5)	44.2 (18.5)	28.5 (12.9)	38.7 (16.7)
	range	(6.4, 51.0)	(10.9, 69.0)	(10.9, 47.6)	(12.6, 65.0)
4	n	19	17	12	12
	mean (SD)	31.9 (17.1)	41.5 (21.6)	25.4 (10.5)	37.5 (21.4)
	range	(8.8, 70.8)	(12.2, 92.1)	(9.2, 42.6)	(10.4, 92.1)
5	n	14	14	12	12
	mean (SD)	28.7 (15.6)	39.7 (16.5)	26.1 (13.0)	38.5 (16.3)
	range	(8.9, 51.9)	(15.6, 63.5)	(9.0, 50.6)	(20.8, 71.5)
6	n	12	13	10	10
	mean (SD)	28.1 (15.7)	39.3 (18.0)	28.8 (14.1)	39.1 (15.8)
	range	(7.4, 52.6)	(11.3, 66.0)	(7.8, 50.2)	(9.9, 63.0)
7	n	10	10	10	10
	mean (SD)	28.3 (21.1)	40.1 (31.2)	29.0 (12.0)	37.1 (17.0)
	range	(8.1, 71.1)	(10.4, 92.1)	(9.8, 46.3)	(11.2, 60.9)
8	n	11	11	10	10
	mean (SD)	26.2 (20.3)	42.8 (31.3)	27.3 (14.1)	36.5 (14.2)
	range	(6.2, 54.6)	(12.1, 92.1)	(9.0, 50.2)	(12.9, 54.6)
9	n	11	11	9	9
	mean (SD)	30.1 (20.1)	40.4 (26.1)	28.5 (14.0)	41.4 (19.5)
	range	(8.8, 60.7)	(11.8, 92.1)	(9.9, 54.6)	(12.4, 64.4)

Table S8. Haemoglobin concentrations in those patients who remained in the ICU.

	Haemoglobin (g/L)									
	Day 0	Day 1	Day 2	Day 3	Day 4	Day 5	Day 6	Day 7	Day 8	Day 9
GM-CSF										
n	17	16	15	15	14	13	11	10	10	10
mean	98.5	95.6	97.2	95.5	96.6	94.9	93.4	88.2	86.9	82.1
sd	15.8	17.8	14.6	16.1	15.4	14.0	16.3	11.9	12.1	15.8
median	102.0	99.0	98.0	98.0	98.5	95.0	91.0	83.5	85.5	81.5
range	(73,126)	(62,127)	(70,124)	(65,121)	(76,120)	(72,121)	(66,121)	(74,105)	(72,107)	(62,110)
IQR	(86,112)	(82.5,107.5)	(88,107)	(82,110)	(83,112)	(88,101)	(81,109)	(78,102)	(75,95)	(67,94)
Placebo										
n	21	21	21	20	20	17	14	15	14	15
mean	99.3	96.3	94.4	92.0	94.1	87.6	87.6	84.2	85.1	90.1
sd	11.0	10.8	12.5	12.8	18.1	13.2	13.0	15.1	15.5	14.8
median	102.0	93.0	91.0	91.5	93.0	85.0	85.0	81.0	82.5	86.0
range	(78, 128)	(76,119)	(79,132)	(76,129)	(71,138)	(71,125)	(72, 119)	(69,125)	(67,123)	(71,123)
IQR	(90,105)	(90,105)	(88,99)	(83.5,95.0)	(81,101.5)	(79,93)	(79,92)	(74,88)	(73,90)	(83,93)

Table S9. Platelet counts for those patients who remained in the ICU.

	Platelet count (x 10^9/L)									
	Day 0	Day 1	Day 2	Day 3	Day 4	Day 5	Day 6	Day 7	Day 8	Day 9
GM-CSF										
n	17	16	15	15	14	12	11	10	10	10
mean	235	193	213	220	241	256	288	329	354	348
sd	221	121	137	144	174	205	227	263	290	323
median	208	202.5	243	206	214	209	207	231	298	258.5
range	(52,999)	(35,425)	(28,509)	(22,509)	(27,596)	(47,713)	(39,752)	(74,873)	(82,979)	(68,1031)
IQR	(97,253)	(87.5,257.5)	(98,279)	(87,290)	(81,299)	(108.5,304)	(105,363)	(173,379)	(157,343)	(117,350)
Placebo										
n	21	21	21	20	20	17	14	15	14	15
mean	172	159	166	171	199	202	228	274	279	320
sd	74	86	90	87	109	112	100	127	117	123
median	164	148	142	166	186.5	179	220	250	289	354
range	(62,309)	(30,324)	(8,328)	(17,370)	(21,421)	(61,425)	(46,410)	(36,491)	(46,453)	(63,477)
IQR	(101,234)	(87,203)	(101,236)	(106.5,213.5)	(116.5,259)	(115,238)	(181,276)	(193,362)	(227,359)	(234,419)

Table S10. Leukocyte counts for those patients who remained in the ICU.

	Leukocytes count (x10^9/L)									
	Day 0	Day 1	Day 2	Day 3	Day 4	Day 5	Day 6	Day 7	Day 8	Day 9
GM-CSF										
n	17	16	15	15	14	13	11	10	10	10
mean	16.2	18.9	20.1	20.2	20.1	18.9	17.3	17.6	17.1	15.9
sd	8.4	7.7	7.8	7.1	9.0	7.6	4.8	4.0	5.0	4.6
median	14.5	19.1	19.1	20.5	18.6	18.2	16.8	18.3	17.9	16.2
range	(4.8,32.9)	(7.7,30.3)	(7.0,35.7)	(10.9,37.0)	(11.2,43.8)	(10.6,41.0)	(8.6,22.7)	(7.7,21.9)	(6.4,24.2)	(7.9,21.1)
IQR	(10.5,21.7)	(12.1,25.3)	(14.0,24.3)	(14.8,24.8)	(13.0,24.6)	(13.7,20.3)	(14.5,22.3)	(16.2,20.1)	(15.2,18.6)	(12.4,20.8)
Placebo										
n	21	21	21	20	20	17	14	15	14	15
mean	11.3	12.6	13.0	13.1	12.9	12.4	13.0	12.8	11.9	11.9
sd	6.1	4.5	6.0	4.7	4.2	4.1	3.6	5.8	5.4	4.9
median	11.9	13.2	11.4	11.7	12.8	12.4	14.0	11.1	11.8	10.1
range	(1.9,29.1)	(4.2,19.6)	(4.5,22.4)	(4.8,22.3)	(4.4,20.5)	(5.1,20.1)	(7.8,17.6)	(5.0,22.9)	(4.3,24.8)	(6.0,21.4)
IQR	(6.0,12.7)	(10.9,15.9)	(9.5,20.2)	(10.0,17.3)	(11.1,15.7)	(8.3,15.6)	(9.2,16.1)	(7.2,16.9)	(8.2,13.8)	(8.2,16.5)

Table S11. Urea concentrations for those patients who remained on the ICU.

	Urea (mmol/L)									
	Day 0	Day 1	Day 2	Day 3	Day 4	Day 5	Day 6	Day 7	Day 8	Day 9
GM-CSF										
n	17	16	15	15	14	13	11	10	10	10
mean	8.29	8.52	9.54	11.27	14.07	16.68	15.59	16.51	17.62	17.81
sd	5.53	7.48	7.94	9.84	12.22	15.11	14.04	13.95	16.77	17.02
median	6.7	5.1	6.7	7.1	9.9	10.0	11.2	9.45	7.7	10.0
range	(1.9,22.0)	(1.5,27.2)	(2.0,30.9)	(2.2,35.8)	(2.5,38.9)	(3.9,51.3)	(4.3,46.3)	(4.9,41.2)	(4.3,45.4)	(4.0,47.0)
IQR	(4.9,9.7)	(4.3,11.0)	(4.4,15.2)	(5.1,13.5)	(5.6,15.6)	(6.0,20.3)	(6.4,26.2)	(6.4,28.7)	(5.4,36.2)	(4.2,37.8)
Placebo										
n	21	21	21	20	20	17	14	15	14	15
mean	12.04	12.49	13.32	13.64	14.24	13.95	14.74	13.19	13.48	11.85
sd	7.33	7.47	6.66	7.26	8.87	9.44	11.25	10.79	10.73	8.41
median	11.6	9.9	12.9	12.85	11.95	11.9	11.5	9.4	11.0	9.4
range	(2.9,30.9)	(2.7,26.9)	(2.2,24.5)	(2.6,29.8)	(3.6,35.9)	(4.0,39.9)	(3.0,43.5)	(3.1,41.5)	(3.4,41.5)	(4.2,33.0)
IQR	(5.9,16.1)	(6.8,18.3)	(8.0,19.1)	(9.4,17.6)	(8.6,18.8)	(8.7,14.2)	(6.8,14.9)	(6.1,13.5)	(7.2,13.8)	(5.4,14.0)

Table S12. Creatinine concentrations in patients who remained on the ICU.

	Creatinine (µmol/L)									
	Day 0	Day 1	Day 2	Day 3	Day 4	Day 5	Day 6	Day 7	Day 8	Day 9
GM-CSF										
n	17	16	15	15	14	13	11	10	10	10
mean	107	110	113	119	137	161	123	131	146	158
sd	68	85	92	102	125	169	113	121	144	151
median	83	70.5	64	65	76	75	69	67.5	80.5	71
range	(40,266)	(44,290)	(40,343)	(38,393)	(35,458)	(36,617)	(36,396)	(38,409)	(36,469)	(33,469)
IQR	(54,146)	(51,157.5)	(51,191)	(54,168)	(49,189)	(57,177)	(51,180)	(47,214)	(41,236)	(42,250)
Placebo										
n	21	21	21	20	20	17	14	15	14	15
mean	130	140	138	135	139	135	146	138	140	134
sd	71	75	73	80	94	87	88	95	88	92
median	132	124	122	121	98.5	112	133	109	112.5	110
range	(59,354)	(56,323)	(51,298)	(49,328)	(53,385)	(50,371)	(49,347)	(44,368)	(42,335)	(43,347)
IQR	(73,153)	(76,171)	(76,176)	(73.5,167)	(74,169)	(75,150)	(73,183)	(61,226)	(88,218)	(58,194)

Table S13. Alanine aminotransferase concentrations in patients who remained on the ICU.

	Alanine aminotransferase (IU/L)									
	Day 0	Day 1	Day 2	Day 3	Day 4	Day 5	Day 6	Day 7	Day 8	Day 9
GM-CSF										
n	17	15	15	13	14	12	10	8	10	9
mean	67	110	95	40	65	51	55	43	53	55
sd	109	204	160	20	89	36	56	20	33	36
median	27	38	44	37	45	38	35	41	39	33
range	(13,449)	(14,812)	(15,646)	(16,85)	(17,365)	(17,137)	(16,206)	(17,85)	(17,114)	(19,120)
IQR	(17,43)	(20,80)	(21,68)	(20,49)	(22,58)	(24.5,75)	(31,59)	(32.5,49)	(31,74)	(29,75)
Placebo										
n	21	20	20	20	18	16	14	14	13	13
mean	83	68	53	47	50	75	97	103	80	58
sd	185	124	78	58	52	110	164	180	106	69
median	27	22.5	20	24	27	23	23	27.5	28	27
range	(10,685)	(11,446)	(12,305)	(11,229)	(12,204)	(13,419)	(12,626)	(10,692)	(13,369)	(11,218)
IQR	(17,34)	(15.5,38)	(16,44)	(15.5,39.5)	(19,70)	(18,82.5)	(17,91)	(22,91)	(17,86)	(16,44)

Table S14. Aspartate aminotransferase concentrations measured in patients who remained on the ICU.

	Aspartate aminotransferase (IU/L)									
	Day 0	Day 1	Day 2	Day 3	Day 4	Day 5	Day 6	Day 7	Day 8	Day 9
GM-CSF										
n	15	15	15	13	11	11	10	8	7	8
mean	99	201	76	31	45	39	34	46	53	52
sd	109	508	140	16	36	31	21	33	44	46
median	71	58	35	27	40	28	29	40.5	43	39.5
range	(19,439)	(24,2030)	(16,574)	(14,70)	(10,135)	(10,102)	(12,84)	(11,118)	(15,144)	(15,155)
IQR	(42,92)	(33,104)	(24,49)	(19,38)	(22,46)	(19,45)	(21,41)	(24.5,56.5)	(17,61)	(20,64)
Placebo										
n	19	17	19	18	18	14	12	12	11	9
mean	151	116	70	61	59	206	258	69	113	42
sd	362	239	94	59	44	513	665	62	168	27
median	34	35	41	36.5	48.5	41.5	41.5	39.5	50	43
range	(15,1617)	(11,1017)	(11,415)	(11,243)	(11,166)	(14,1973)	(13,2360)	(16,226)	(14, 578)	(10,90)
IQR	(26,108)	(24,79)	(20,64)	(23,83)	(24,76)	(20,166)	(23.5,112.5)	(25,101.5)	(19,118)	(16,62)

Figure S1. Monocyte HLA-DR illustrated as individual points.

Monocyte HLA-DR was quantified by flow cytometry using a commercial Quantibrite kit at day 0, ie baseline, before treatment, day 2, day 4/5, day 6/7 and day 8/9.

Figure S2. Circulating cytokine measurements.

Cytokine concentrations were determined by cytometric bead array (Becton Dickinson). (A) IL-6, (B) IL-8, (C) IL-1β, (D) IL-10, (E) TNFα, (F) GM-CSF and (G) IL12-p70. White columns = placebo, dark columns = GM-CSF.

GM-CSF: Statistical Analysis Plan for RCT

Full title (Overall study): DOES GM-CSF RESTORE EFFECTIVE NEUTROPHIL FUNCTION IN CRITICALLY ILL PATIENTS?

Objectives

(Overall study): To test whether clinical administration of GM-CSF replicates the effects seen *ex vivo*, in the blood of critically ill patients, by restoring neutrophil phagocytosis.

Trial arms: 2 groups defined by treatment. Recruitment of 19 patients per group was intended with random allocation by randomised block randomisation used to allocate treatment:

Group 1: GM-CSF
Group 2: Placebo

Intervention group was blinded to both investigators and participants excepting that trial monitoring was conducted on an unblinded basis. Unblinding will take place immediately prior to analysis of the data.

Outcome measures:
Primary:

- Neutrophil phagocytic capacity 2 days after administration of GM-CSF/placebo (as measured by the percentage of neutrophils ingesting ≥ 2 zymosan particles ex vivo).

Secondary:
Biological measures:

- Sequential neutrophil phagocytic capacity (to determine sustainability of any observed effects in the primary endpoint)
- Neutrophil phagocytic capacity measured as 'area under the curve' over the study period. Other assessments of neutrophil function:
    - Reactive oxygen species (ROS) generation (continuous measure)
    - Distances migrated on a chemotaxis assay (change from baseline)
    - Apoptotic rate (proportion of cells identified as apoptotic by flow cytometric criteria, and/or by morphological criteria)
- Sequential monocyte HLA-DR expression.
- If available; serum/plasma measures of inflammatory and anti-inflammatory mediators. It will be recorded whether unblinding of patients took place before this data was available for analysis.

Clinical Measures:

- Baseline clinical and demographic measurements. The following will be summarised by group but not subject to further analysis:
    - Age
    - Gender
    - Weight
    - Apache II score (at admission)
    - Smoking status

- Dichotomous co-morbidity indicators for:
    - Arthritis
    - Osteoporosis
    - Asthma
    - COPD
    - ARDS
    - Emphysema
    - Angina
    - Congestive heart failure
    - MI
    - Neurological disease
    - Cerebrovascular disease
    - Peripheral vascular disease
    - Diabetes
    - Upper GI disease
    - Depression
    - Anxiety/panic disorders
    - Visual impairment
    - Hearing impairment
    - Obesity
    - Degenerative disc disease

- Sequential organ failure assessment (SOFA) score (to be treated as an ordinal outcome; change from baseline to day 7 (3 days after final dose) will also be considered in this way)
- Length of stay in ICU
- The incidence of ICUAIs (as defined by Hospitals in Europe Link for Infection Control Surveillance (HELICS) criteria)
- All cause mortality 30 days post randomisation
- Number of days of mechanical ventilation

Safety measures:

- Full blood count – including haemoglobin level and platelet count
- White cell count including neutrophil, monocyte, eosinophil and lymphocyte counts
- Urea and electrolytes (sodium, potassium, urea, creatinine) and liver function tests (alkaline phosphatase, gamma glutamyl transferase, alanine aminotransferase, aspartate aminotransferase)

Serious adverse events (SAEs) and occurrence of suspected unexpected serious adverse reactions (SUSARs) will also be recorded but not subject to formal analysis beyond tabulation by group.

Analysis: As this is a phase 2 clinical study, with relatively small numbers of participants, we will principally report descriptive statistics. Data for the outcomes stated above will be summarised within each dosing group using descriptive statistics (means and standard deviations for continuous data and frequencies, medians and interquartile ranges for ordinal data, proportions/rates for dichotomous/categorical data). Confidence intervals will also be presented as appropriate.

Summaries of demographic and operational information (such as number of withdrawals) will also be reported in a similar fashion. The change in the primary outcome measure between baseline and the primary outcome point of 2 days after treatment administration as a patient level outcome will also be considered.

We will also explore comparative analysis of the primary endpoint (neutrophil phagocytic capacity at 2 days after administration of GM-CSF/placebo) between the treatment groups although, due to the sample size, this will be exploratory in nature rather than definitive.

The analysis groups will be compared for the primary outcome using a 2-sample t-test. Should data be considered to be non-normal the alternative non-parametric test (Mann-Whitney) will be used. With the small sample size, normality will be assessed graphically via probability plots & histograms and by consideration of the mean and median values.

Additional analysis will be performed using Analysis of Covariance (ANCOVA) in order to adjust for the effects of site (the stratification factor) and the baseline value of the outcome measure (if appropriate). Should a potentially clinically and statistically significant difference between groups be observed, consideration will be given to additionally adjusting for baseline clinical and demographic covariates. However, as this analysis is exploratory in nature given the sample size of the study it is not likely that the model will support additional covariates.

This measure will also be analysed using ANCOVA in the above manner at the following time points: Baseline/day 0 (without the baseline adjustment), day 2, day 4/5, day 6/7 & day 8/9.

Again, however this analysis can only be considered exploratory due to the small sample size.

Secondary endpoints (other than baseline clinical and demographic measures as outlined, the SOFA score and safety measures described below) will be examined using similar methods with consideration given to comparative analyses as described for the primary outcome. However, dichotomous variables would be analysed in an exploratory fashion using either chi-squared or Fisher's exact tests (in place of the t-test) and logistic regression in place of ANCOVA to adjust for the effects of site and baseline values. As for the primary outcome no further covariates will be considered and these analyses would be considered to be exploratory in nature. The neutrophil phagocytic capacity will be considered in any comparative analysis at fixed time points only as described earlier.

Group membership will initially be defined on an Intention to treat basis. Following the primary ITT analysis as described above, further sub-group analysis will be performed by separate consideration of those who received at least two doses of treatment and those who received fewer. Additionally, length of stay in ICU, incidence of ICUAIs & number of days of mechanical ventilation will be summarised separately by group for those patients surviving to the end of the trial.

Statistical significance, where appropriate, will be set at 0.05.

Safety data:

Although the incidence of ICUAIs and 30-day mortality will be documented and descriptive statistics presented, these important clinical endpoints are not included as outcome variables in the data

analysis as this proof of concept study is not adequately powered to assess these. Other safety measures, other than AE and SAE numbers, may be subject to comparative analysis as described for the secondary outcome measures above.

Missing data:

Data with missing observations (other than due to mortality) will be examined to determine both the extent of and reason for such omissions. Any missing data will be described. The use of multiple imputation techniques may be considered for the primary outcome alone should data be missing on the primary outcome measure for participants completing the study to a sufficient extent (approximately >10%) but given the exploratory nature of this study it is unlikely that this approach would be felt to be informative.

Software: Unless otherwise stated analyses will be carried out using the following computer software (versions and attributions to be recorded at time of use):

- Stata (as first choice software).
- Other software if considered appropriate (use to be recorded).
