# Supplementary figures and images for: Randomised controlled trial of GM-CSF in critically ill patients with impaired neutrophil phagocytosis
Source: Thorax. 2018 Jul 31;73(10):918–25. doi: 10.1136/thoraxjnl-2017-211323 (PMC6166597; doi:10.1136/thoraxjnl-2017-211323)

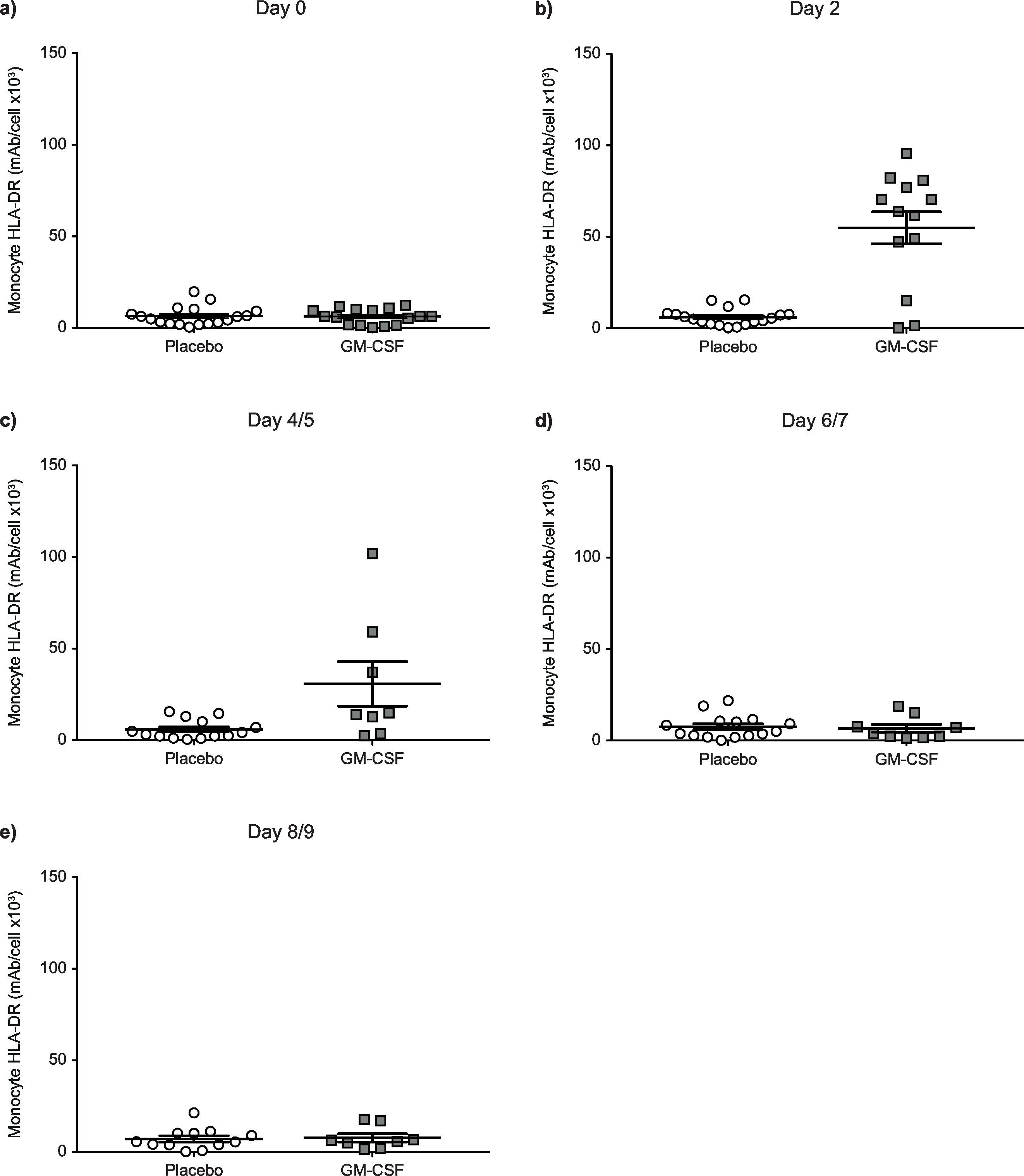

Supplement: Supplementary data [file thoraxjnl-2017-211323supp002.jpg]

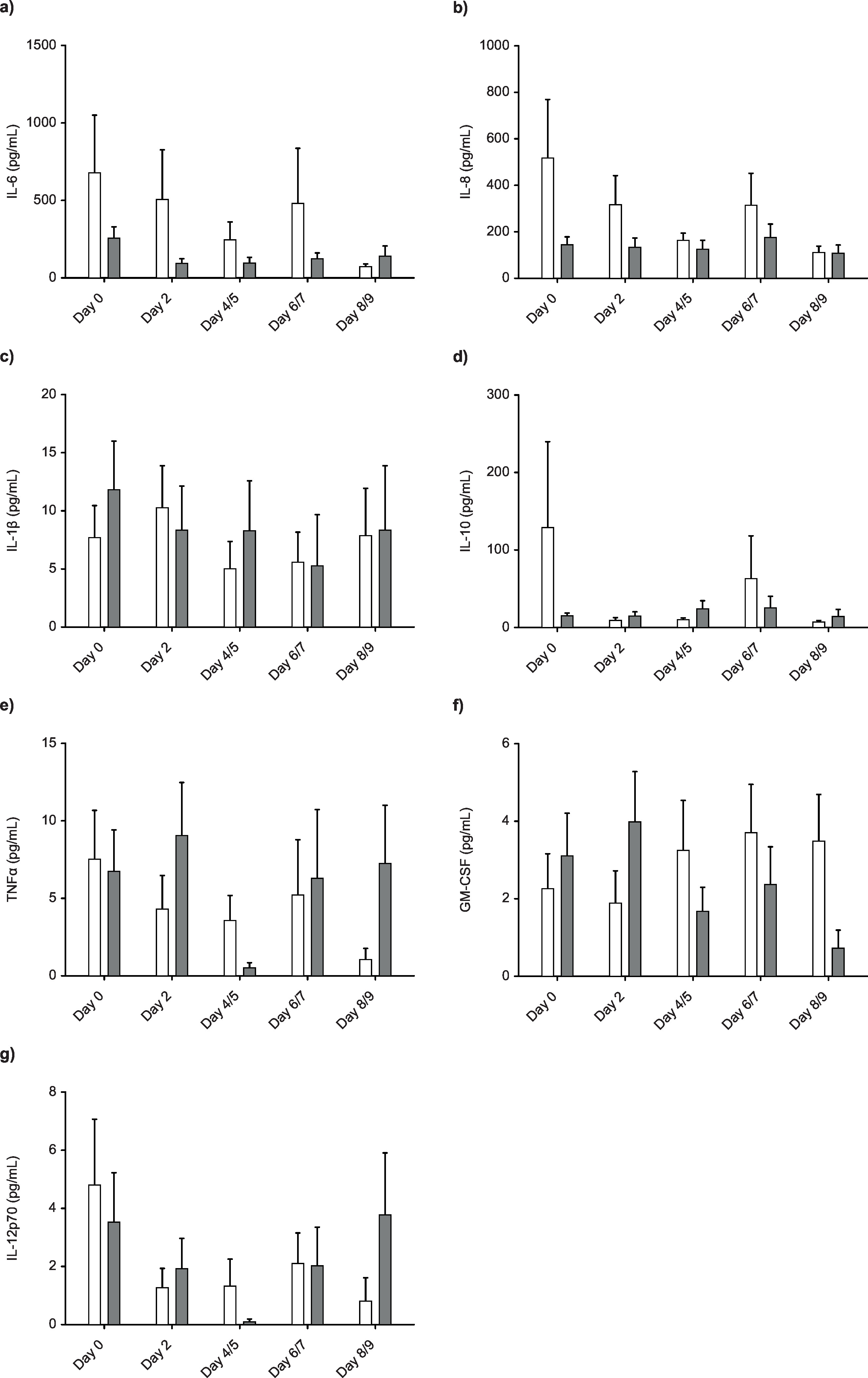

Supplement: Supplementary data [file thoraxjnl-2017-211323supp003.jpg]
